# The Lung Microbiome Modulates Pain‐Like Behavior Via the Lung–Brain Axis in a Nitroglycerin‐Induced Chronic Migraine Mouse Model

**DOI:** 10.1002/advs.202416348

**Published:** 2025-03-31

**Authors:** Biying Liu, Chengya Huang, Xin Li, Haonan Yu, Yuefeng Xia, Kun Liu, Xingji You, Jingxiang Wu

**Affiliations:** ^1^ Department of Anesthesiology Shanghai Chest Hospital Shanghai Jiao Tong University School of Medicine Shanghai 200030 China; ^2^ School of Medicine Shanghai University Shanghai 200444 China

**Keywords:** lung microbiome, lung‐brain axis, migraine

## Abstract

Chronic migraine is one of the most common pain disorders, characterized by significant disability and a lack of safe, long‐term, and effective treatment options. Recent studies highlight the interaction between the lung microbiota and the central nervous system. In this study, a nitroglycerin (NTG)‐induced chronic migraine model is constructed in male C57BL/6 mice to explore these interactions. Notable alterations are observed in the lung microbiota of migraine‐afflicted mice. Notably, there is a marked decrease in Proteobacteria in the chronic migraine group, associated with short‐chain fatty acids and 5‐hydroxytryptamine (5‐HT). After the intratracheal injection of neomycin, the diversity of the lung microbiota is altered, resulting in the relief of migraines. This effect is also observed in mice that receive neomycin‐treated bronchoalveolar lavage fluid (BALF) transplantation, further demonstrating the role of lung microbiota in this process. The altered lung microbiota activate the pulmonary vagus nerve via the Brain‐derived neurotrophic factor‐tropomyosin receptor kinase B (BDNF‐TrkB) pathway in the lung, which projects to the central nucleus of the solitary tract (NTS) and the dorsal raphe nucleus (DRN). This activation, in turn, stimulates the 5‐HT neurons in the DRN, resulting in increased serotonin levels that contribute to pain relief in the chronic migraine model.

## Graphical abstract

Intratracheal neomycin alters pulmonary microbiota, activating the vagus nerve via lung brain‐derived neurotrophic factor/tropomyosin receptor kinase B (BDNF/TrkB) pathway, further projecting to nucleus of solitary tract (NTS), then dorsal raphe nucleus (DRN), where activated serotoninergic neurons increase brain serotonin, alleviating migraine. This Figure is drawn with Figuredraw (www.Figuredraw.com).

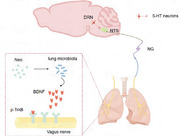

## 1 Introduction

Migraine is a chronic neurovascular disorder and a leading cause of disability,^[^ ^1^, ^2^ ^]^ affecting ≈15% of the global population;^[^ ^3^, ^4^ ^]^ while chronic migraine has a prevalence of 2%.^[^ ^5^ ^]^ It is typically characterized by recurrent episodes of severe headaches, often accompanied by nausea, vomiting, and other physiological or psychological symptoms.^[^ ^6^, ^7^, ^8^, ^9^ ^]^ Patients with chronic migraine have a larger burden on medical expenses and more comorbidities and are more resistant to treatment than acute migraine. Several treatments are available for acute attacks of chronic migraine with robust evidence supporting their efficacy, including analgesics and nonsteroidal antiinflammatory drugs (NSAIDs) for mild to moderate attacks, triptans, and opioids for severe attacks.^[^ ^10^, ^11^ ^]^ Antidepressants, antiepileptic drugs,^[^ ^12^ ^]^ and emerging therapies such as Ubrogepant,^[^ ^13^ ^]^ a small‐molecule calcitonin gene‐related peptide (CGRP) receptor antagonist,^[^ ^14^, ^15^ ^]^ are used for preventive treatment of chronic migraine. However, these medications generally provide only transient relief and are associated with side effects, the potential for drug abuse, and unsatisfactory treatment outcomes.^[^ ^16^, ^17^ ^]^ Despite significant advances in understanding the pathophysiology of migraine, its underlying mechanisms remain poorly understood, leading to a lack of effective treatments^.^ Therefore, the development of effective migraine therapies continues to be a primary focus of current research.

Recent studies have revealed that migraine headaches are associated with an increased risk of respiratory disorders, such as asthma and bronchitis.^[^ ^18^ ^]^ Further, migraine‐like headaches have been reported in patients with lung carcinoma.^[^ ^18^ ^]^ In addition, recent research has discovered the existence of a lung–brain axis, where the lung microbiome interacts with the brain.^[^ ^19^ ^]^ Previous studies have indicated that the gut microbiome significantly impacts migraines and headaches.^[^ ^20^, ^21^, ^22^, ^23^ ^]^ The administration of antibiotics can induce microbiome dysbiosis and alter host–bacteria interactions,^[^ ^24^ ^]^ and gut microbiome dysbiosis has been shown to trigger migraine‐like pain in mouse models.^[^ ^22^ ^]^ These findings suggest that specific communications between the lung microbiome and migraines may exist, warranting further exploration.

Among the proposed theories for the onset and progression of migraines, the activation of the trigemino‐vascular system^[^ ^25^ ^]^ most comprehensively reflects the underlying pathophysiological processes^[^ ^8^ ^]^ and has gradually emerged as a mainstream mechanism. During migraine onset and progression, the classic ascending pain conduction pathway is specifically the trigeminal ganglion (TG)‐trigeminal nucleus caudalis (TNC)‐thalamus,^[^ ^25^, ^26^, ^27^ ^]^ as well as the descending pain modulation system in the brainstem. This descending system is primarily composed of neurons located in the periaqueductal gray matter (PAG),^[^ ^27^, ^28^ ^]^ the medial ventral part of the medulla oblongata (RVM),^[^ ^28^, ^29^ ^]^ and a portion of the dorsolateral pons.^[^ ^30^ ^]^ The descending pathway contains a significant number of serotonergic neurons, which are crucial for descending pain modulation;^[^ ^31^ ^]^ these serotonergic fibers can project to various structures within the trigemino‐vascular system.^[^ ^32^ ^]^ Therefore, our research focuses on these two pathways, examining changes in TNC tissue within the migraine model and alterations of 5‐HT neurons in the PAG.

Here, we investigate the relationship between the lung microbiome and migraine in a NTG‐induced chronic migraine mouse model, focusing on the underlying analgesic effects of lung microbiome and its mechanisms. We find that microbiome dysbiosis caused by intratracheal administration of neomycin alleviates pain‐like behaviors associated with migraine by activating vagal sensory neurons innervating the lungs, which project to the nucleus tractus solitarius (NTS), and subsequently, promote the release of 5‐hydroxytryptamine (5‐HT) to relieve migraine symptoms. Overall, this study demonstrates the role of the lung microbiome in regulating the central nervous system and suggests a potential therapeutic strategy for migraine treatment via the lung–brain axis in the NTG‐induced migraine model.

## 2 Results

### 2.1 Manipulations of Lung Microbiome Can Relieve the Pain in a Migraine Model

**Figure 1:**
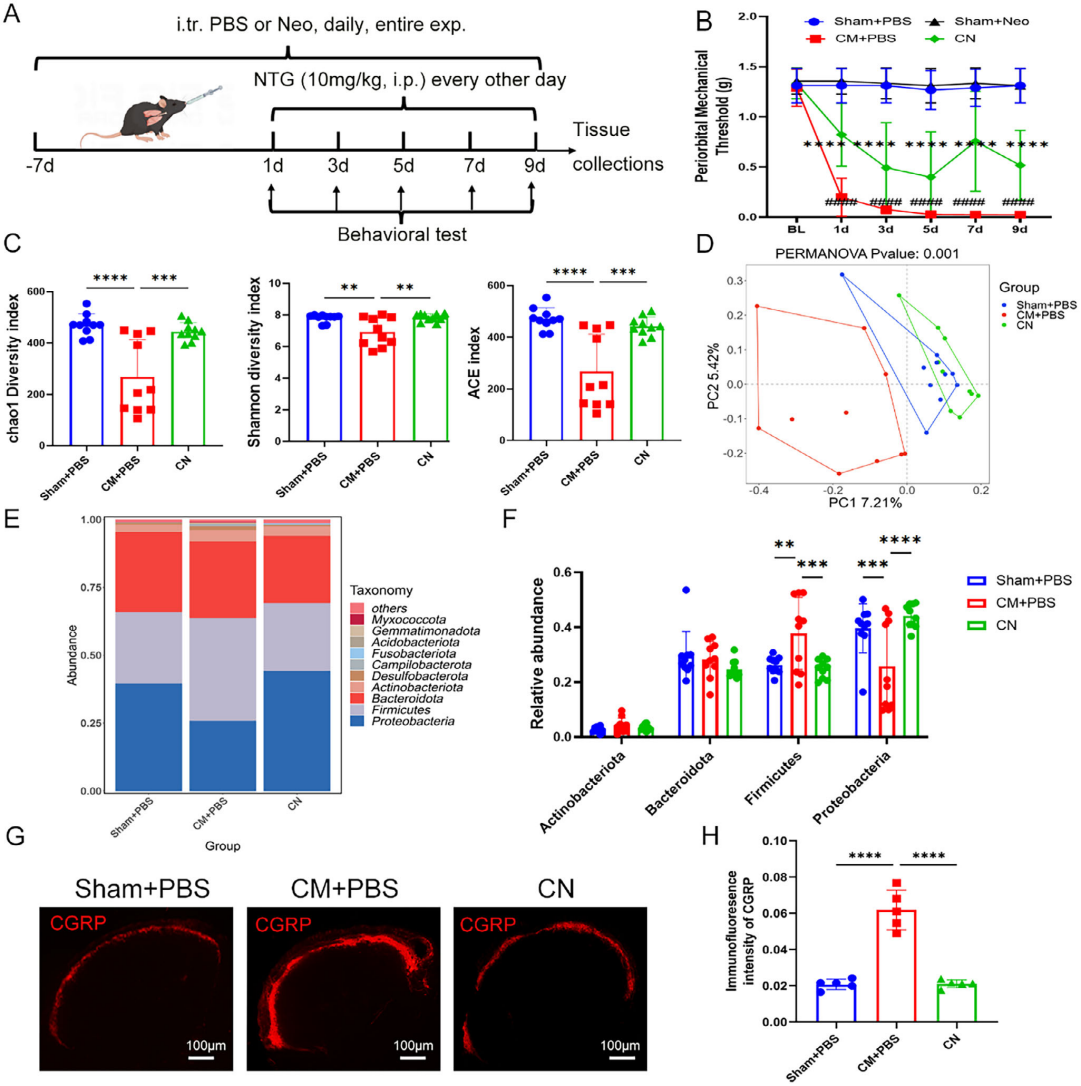
Neomycin‐induced alterations in the lung microbiome alleviate migraine‐related pain in a chronic migraine mouse model. A) Schematic of the experimental timeline. Neo, neomycin; NTG, nitroglycerin. B) The periorbital mechanical threshold of sham+PBS, sham+Neo, CM+PBS, and neomycin‐treated chronic migraine (CN) group. *n* = 6 per group. *, CM versus CN; #, Sham+PBS versus CM. C) Chao1, Shannon, and ACE index. *n* = 10 (all groups). D) Principal co‐ordinate analysis of Bray distance. E,F) Average relative abundance of the bacterial phylum of the lung microbiota. G,H) Representative images (G) and quantification (H) of CGRP expression in the TNC tissue after neomycin treatment. Scale bar: 100 µm. *n* = 5. Values are presented as mean ± SEM. *p < 0.05, **p < 0.01, ***p < 0.001, and ****p < 0.0001. One‐way ANOVA test or two‐way ANOVA test was used for multivariate analysis.

The lung is characterized by a unique environment that harbors a distinct microbial flora,^[^ ^33^ ^]^ which play a crucial role in regulating local immune responses during pathological processes such as asthma,^[^ ^34^ ^]^ idiopathic pulmonary fibrosis, and tumors,^[^ ^35^ ^]^ some of which have been reported to be associated with migraine attacks. We aimed to investigate whether lung microbiome influences migraine attacks. To this end, we constructed a NTG‐induced chronic migraine (CM) model. In the migraine group, we observed a significant alteration in lung microbiome diversity, with a reduced number and variety of microbiota compared to the sham group (Figure S1A,B, Supporting Information). Daily intratracheal administration of 0.2 mg neomycin from −7 day to the end of the experiment (**Figure** **1**A) was used to induce notable changes in the microbiome. Importantly, in the neomycin‐treated migraine group (CN), the periorbital mechanical threshold was elevated (Figure 1B). In contrast, mice in the CM group without neomycin treatment exhibited a lower mechanical threshold; while, treatment with PBS or neomycin did not affect the periorbital mechanical threshold in the sham group (Figure 1B). Neomycin treatment significantly altered the lung microbial environment, increasing both the quantity and diversity of microbiota (Figure 1C,D; Figure S1C, Supporting Information). The Shannon, chao1, and ACE index in the CM group were lower than those in the sham and neomycin‐treated migraine group (CN) group, indicating a significant decrease in species diversity after NTG injection; while, neomycin treatment restored microbial diversity in numbers and species (Figure 1C). Principal component analysis (PCoA) reflected a significant separation between three groups (Anosim, *p* = 0.001). Moreover, at the phylum level, NTG injection markedly reduced the relative abundance of proteobacteria (25.8%) and increased the relative abundance of Firmicutes (37.8%); while, neomycin administration oppositely increased the relative abundance of proteobacteria to 44.2% and decreased the relative abundance of Firmicutes to 25.0% (Figure 1E,F).

**Figure 2:**
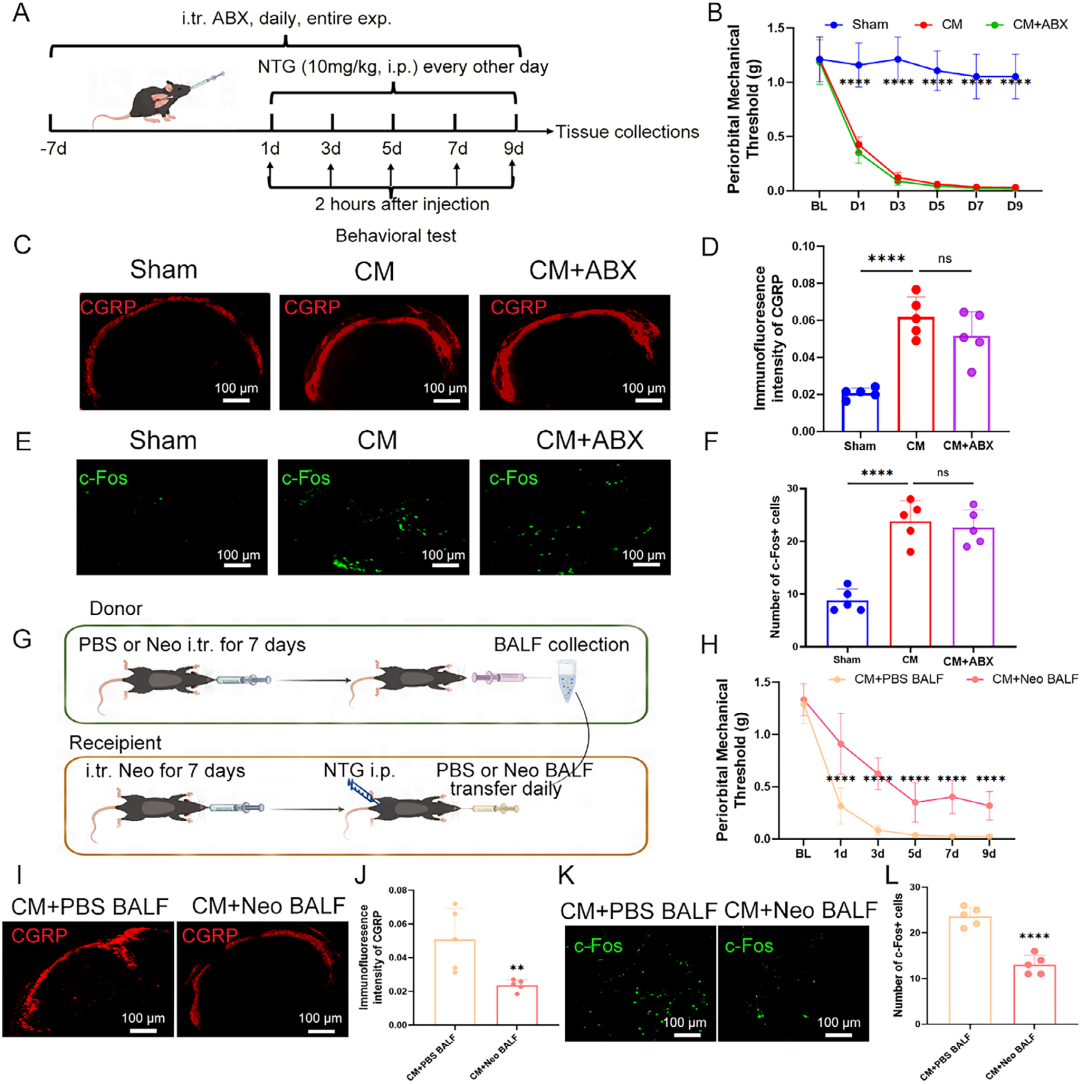
ABX injection does not alleviate migraine pain; while, lung microbiota transfer exhibits analgesic effects. A) Schematic of the experimental timeline. ABX denotes broad‐spectrum antibiotics. B) The periorbital mechanical threshold of sham, CM (chronic migraine), and CM+ABX group. *n* = 6 per group. C,D) Representative images (C) and quantification (D) of CGRP expression in the TNC tissue. Scale bar: 100 µm. *n* = 5. E,F) Representative images (E) and quantification (F) of c‐Fos expression in the TNC tissue. Scale bar: 100 µm. *n* = 5. G) Schematic of the lung microbiota transplantation experiment. Microbiota isolated from bronchoalveolar lavage fluid (BALF) of neomycin‐ or PBS‐treated mice were intratracheally transferred to recipient mice. H) Periorbital mechanical thresholds in mice receiving microbiota transfers from neomycin‐treated (CM+Neo BALF) versus PBS‐treated (CM+PBS BALF) donors (*n* = 6 per group). I,J) Representative images (I) and quantification (J) of CGRP expression in the TNC tissue of mice receiving microbiota transfers from neomycin‐ or PBS‐treated donors. Scale bar: 100 µm. *n* = 5. K,L) Representative images (K) and quantification (L) of c‐Fos expression in the TNC tissue of mice receiving microbiota transfers from neomycin‐ or PBS‐treated donors. Scale bar: 100 µm. *n* = 5. Values are presented as mean ± SEM. *p < 0.05, **p < 0.01, ***p < 0.001, and ****p < 0.0001. One‐way ANOVA test or two‐way ANOVA test was used for multivariate analysis. Unpaired *t*‐tests were used for the comparison of two groups.

CGRP is known to play a role in the onset and persistence of migraines. Immunostaining revealed a decreased expression of CGRP in the neomycin‐treated group compared to the migraine group (Figure 1G,H) in the TNC region. The number of c‐Fos+ neurons, an indicator of neuronal activation, in the TNC region was reduced in the neomycin‐treated migraine group relative to the CM group (Figure S1D,E, Supporting Information). Further, the presence of the lung microbiome was essential for the neomycin effect; intratracheal application of broad‐spectrum antibiotics (ABX), which deplete lung microbiota, did not influence migraine (**Figure** **2**A–F). The ABX treatment may not have completely eradicated all bacterial taxa; the treatment significantly reduced the overall microbial diversity. This aligns with previous reports indicating that ABX treatment results in bacterial depletion; although, some bacterial species may persist in low numbers.^[^ ^36^, ^37^ ^]^ In contrast, neomycin treatment selectively altered the microbial composition by notably increasing Proteobacteria and decreasing Firmicutes. These findings suggest that the analgesic effects observed with neomycin are more likely due to specific shifts in the microbiome rather than a broad depletion of the microbiota, as seen with ABX. The alterations in pain threshold and CGRP expression in TNC tissue suggest that we successfully constructed a reliable chronic migraine model and that inhalation of neomycin can alleviate pain.

To confirm the role of the lung microbiome in regulating hyperalgesia in CM model, we conducted a lung bacterial transplant experiment as a substitute for direct neomycin treatment (Figure 2G). We transferred microbiota isolated from bronchoalveolar lavage fluid (BALF) collected from mice treated with either PBS or neomycin following the cessation of intratracheal neomycin treatment. Notably, mice receiving BALF microbiota from neomycin‐treated donors (CM+Neo BALF) exhibited a significantly higher periorbital mechanical threshold compared to those receiving PBS‐treated BALF (CM+PBS BALF) (Figure 2H). Consistent with this, the expression of CGRP and c‐Fos was elevated in the CM+ Neo BALF group compared to the CM+ PBS BALF group (Figure 2I–L; Figure S1D,E, Supporting Information). These data indicate that manipulation of the lung microbiome influences pain relief in the migraine model, revealing a connection between the lung microbiome and the central nervous system (CNS).

### 2.2 Activation of 5‐HT Neurons in the Dorsal Raphe Nucleus (DRN) Following Lung Microbiome Manipulation

**Figure 3:**
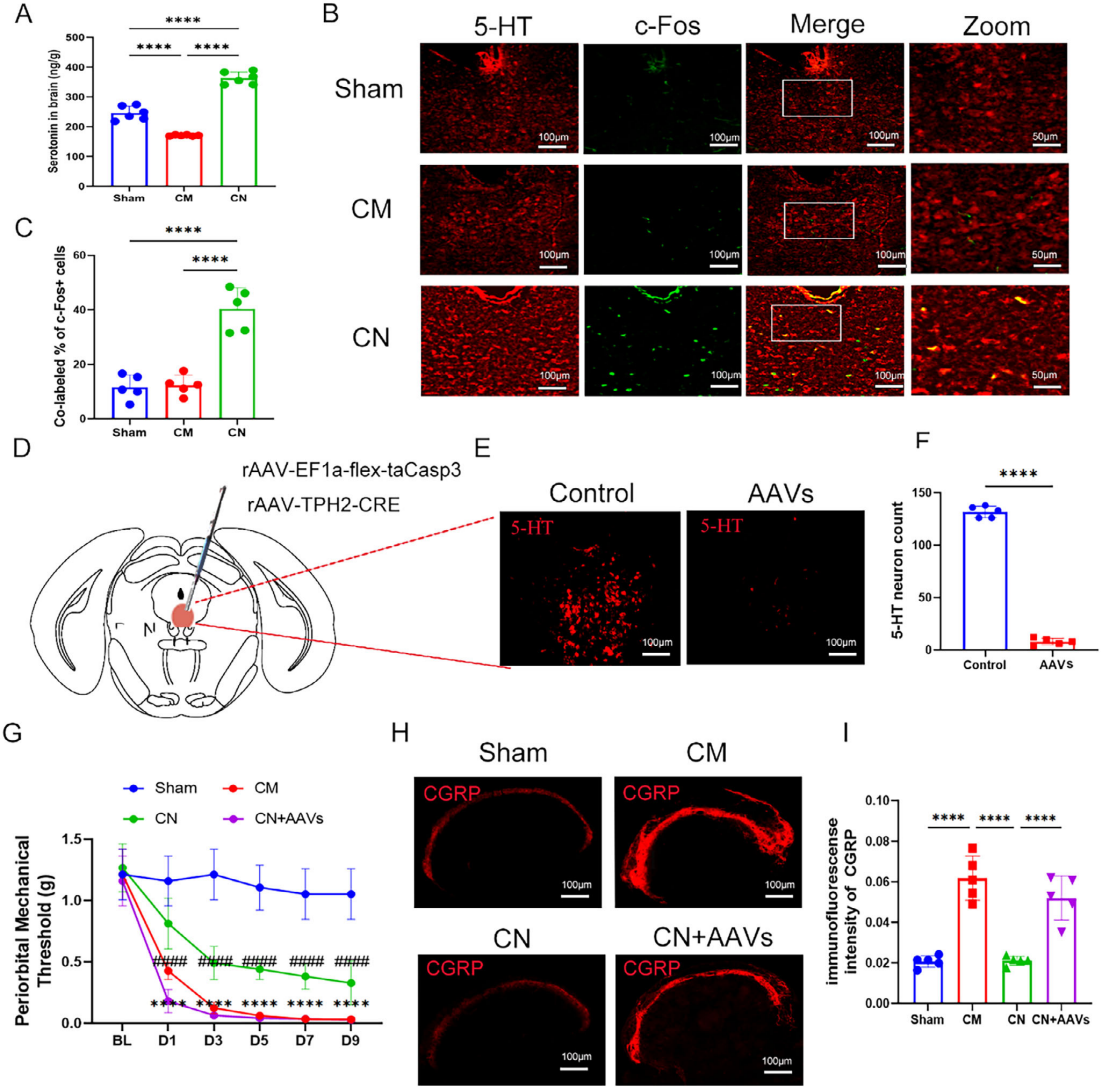
Activation of 5‐HT DRN neurons is essential for the analgesic effects of lung microbiome manipulation. A) The 5‐HT levels in the brains of sham, chronic migraine (CM), and neomycin‐treated chronic migraine (CN) groups. *n* = 6 per group. B,C) Representative images (B) and quantification (C) of 5‐HT and c‐Fos colocalization. Scale bar: 100 µm. *n* = 5. D) The experimental procedure of AAVs‐mediated ablation of 5‐HT neurons in the DRN. E,F) Representative images (E) and quantification (F) showing the elimination of 5‐HT neurons in the DRN. Scale bar: 100 µm. *n* = 5. G) The periorbital mechanical threshold of sham, chronic migraine (CM), and neomycin‐treated chronic migraine (CN) and neomycin‐treated chronic migraine with AAVs injection for ablation (CN+AAVs). *n* = 5 per group. H,I) Representative images (H) and quantification (I) of CGRP expression in the TNC tissue, Scale bar: 100 µm. *n* = 5. Values are presented as mean ± SEM. *p < 0.05, **p < 0.01, ***p < 0.001, and ****p < 0.0001. One‐way ANOVA test or two‐way ANOVA test was used for multivariate analysis. Unpaired *t*‐tests were used for the comparison of two groups.

Serotonin (5‐hydroxytryptamine, 5‐HT) is involved in the pathophysiology of migraine. First, we found that the serotonin levels in the brains of CM mice were significantly reduced. Neomycin treatment reversed this reduction, nearly doubling serotonin levels compared to the migraine group. (**Figure** **3**A). To verify whether neomycin induced 5‐HT neurons activation, we treated TRAP2; Ai9 mice, a genetically labelled TRAPed neurons with a Cre‐dependent tdTomato reporter, with PBS or neomycin for 7 days (Figure S2A, Supporting Information). Examination of several brain regions associated with pain revealed an increase in TRAPed neurons in the primary somatosensory cortex (S1), DRN, and NTS in the neomycin‐treated group (Figure S2B,C, Supporting Information). Given that the DRN is the primary source of 5‐HT neurons in the brain, we hypothesized that neomycin treatment may activate 5‐HT neurons in this region. Subsequent analysis confirmed in the DRN following neomycin treatment (Figure 3B). As anticipated, there was an increased colocalization of c‐Fos and 5‐HT in the DRN following neomycin injection (Figure 3B,C). Collectively, these data indicate that 5‐HT^DRN^ neurons were activated by intratracheal neomycin infusion.

To investigate the potential role of 5‐HT^DRN^ neurons in communication between lung microbiota and the brain in the CM model, we initially constructed a serotonergic neuron‐lesioned mouse model through a two‐virus stereotaxic injection into the DRN to selectively ablate 5‐HT^DRN^ neurons (rAAV‐EF1a‐flex and rAAV‐TPH2‐CRE) (Figure 3D). Three weeks post‐surgery, the mice underwent neomycin treatment and NTG injection as outlined in the Figure. The viral injection resulted in a significant ablation of 5‐HT^DRN^ neurons (Figure 3E,F), which did not alter the mechanical threshold prior to neomycin and NTG administration. However, neomycin treatment did not effectively increase the periorbital mechanical threshold in neomycin‐treated CM mice subjected to 5‐HT^DRN^ neuron ablation using AAVs (Figure 3G). Similarly, neomycin did not suppress the expression of CGRP in the TNC region following viral ablation (Figure 3H,I). In addition, to further investigate whether the activation of 5‐HT neurons induced by neomycin‐mediated alteration in lung microbiota is essential for its analgesic effects, the AAV‐DIO‐hM4Di virus was injected into the DRN of Sert‐Cre mice and CNO was administered through the drinking water to chronically silence 5‐HT neurons after neomycin pre‐treatment (Figure S3A, Supporting Information). We found that neomycin treatment did not improve the periorbital mechanical threshold in the neomycin‐treated migraine group of mice with hM4Di silencing of 5‐HT^+^ neurons (Figure S3B,C, Supporting Information). In conclusion, specific lung microbiota exerted analgesic effects through 5‐HT^DRN^ neurons in the CM model.

### 2.3 Lung Microbiota Activates 5‐HT^DRN^ Neurons Through Lung‐Innervating Sensory Neurons

**Figure 4:**
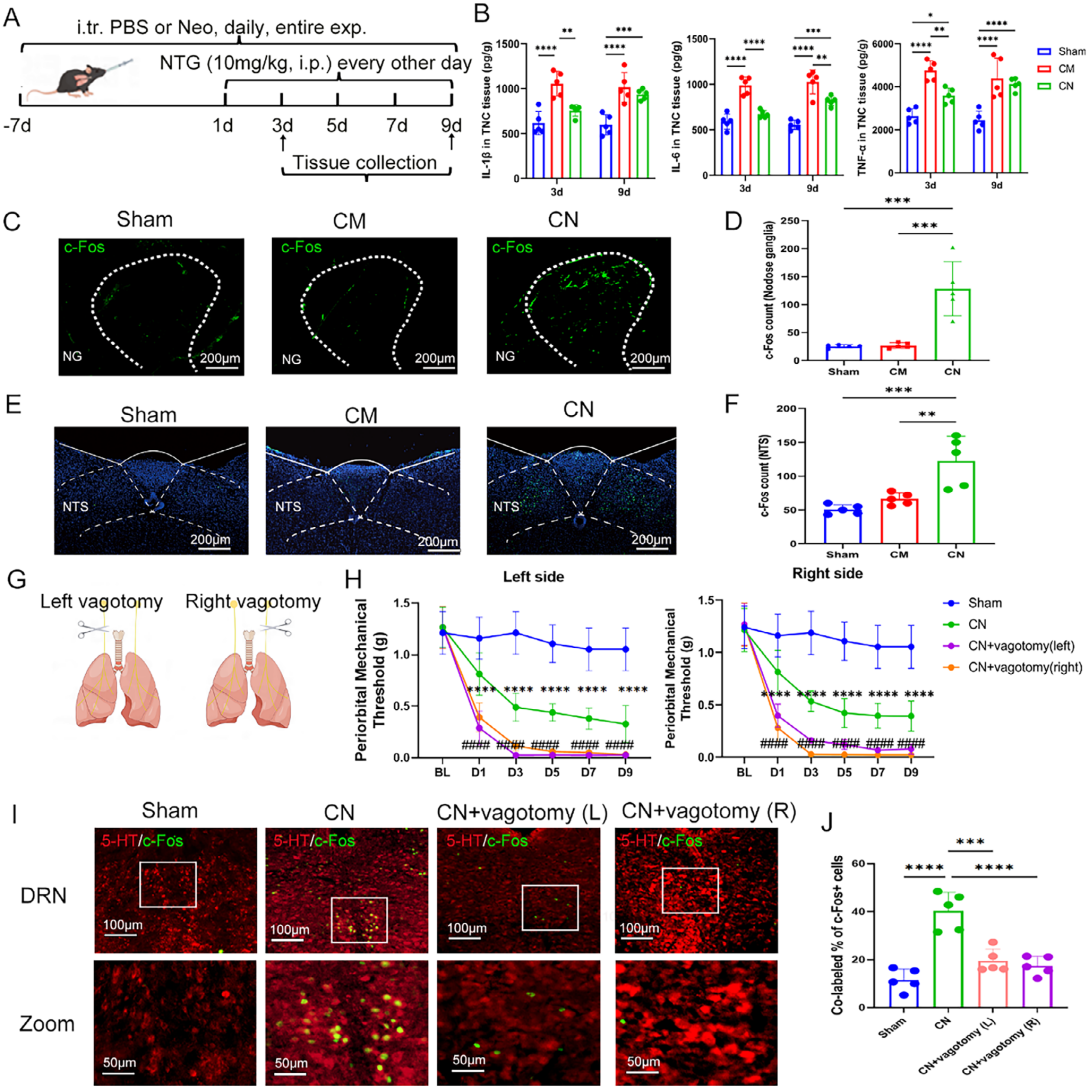
The impact of lung microbiota on migraine is achieved through the activation of the vagus nerve, projecting to the NTS. A) Schematic of the experimental timeline. B) ELISA‐quantified serum concentration of IL‐1β, IL‐6, and TNF‐α. *n* = 5. C,D) Representative images (C) and quantification (D) of c‐Fos expression in the NG, Scale bar: 200 µm. *n* = 5. E,F) Representative images (E) and quantification (F) of c‐Fos expression in the NTS, Scale bar: 200 µm. *n* = 5. G) The experimental procedure of unilateral vagotomy. H) The left and right periorbital mechanical threshold. *n* = 6 per group. * neomycin‐treated chronic migraine (CN) versus CN+ vagotomy(left); # neomycin‐treated chronic migraine (CN) versus CN+ vagotomy(right). I,J) Representative images (I) and quantification (J) of 5‐HT and c‐Fos colocalization. Scale bar: 100 µm. *n* = 5. Values are presented as mean ± SEM. *p < 0.05, **p < 0.01, ***p < 0.001, and ****p < 0.0001. One‐way ANOVA test or two‐way ANOVA test was used for multivariate analysis.

Several studies have confirmed that gut microbiota can activate peripheral projection neurons, facilitating interactions with the central nervous system through neuroimmune responses,^[^ ^38^ ^]^ direct vagus nerve activation,^[^ ^39^ ^]^ and microbiome metabolism.^[^ ^40^ ^]^ To identify the primary mechanism underlying the lung microbiome–brain interaction, we first collected TNC tissue at 3 and 9 d, representing the early and late stage of migraine and measured the levels of tumor necrosis factor‐α (TNF‐α), Interleukin‐ 6 (IL‐6), and Interleukin‐ 1β (IL‐1β) (**Figure** **4**A). The results indicated that at 3d, the neomycin treatment group exhibited an anti‐inflammatory effect (Figure 4B). At 9 d, although the IL‐6 level increased, it remained lower in the neomycin‐treated chronic migraine group; while, the levels of TNF‐α and IL‐1β in the neomycin treatment group were not significantly different from those in the CM group (Figure 4B). Neomycin exhibits anti‐inflammatory effects only during the early stages of treatment. However, its migraine‐relieving effects persist from the initial phase through the later stages. Therefore, this suggests that the alleviation of migraine by neomycin may not be mediated by its anti‐inflammatory in the TNC region.

Considering the significant distribution of vagal nerve innervation within the lungs, we aimed to investigate whether alterations in the lung microbiota could activate pulmonary‐innervating sensory neurons. To this end, we collected nodose ganglia, where the cell bodies of vagal sensory neurons are located. We observed notable c‐Fos activation in the nodose ganglia of the neomycin injection group compared to the Sham and CM groups (Figure 4C,D), confirming the activation of lung‐innervating sensory neurons.

As vagal afferents predominantly project to the NTS in the medulla, we observed an increase in c‐Fos expression in the NTS following neomycin infusion (Figure 4E,F). To further investigate the role of the vagus nerve in the interaction between lung microbiota and the CNS, we conducted unilateral vagotomy before neomycin infusion (Figure 4G). Notably, the antalgic effect of neomycin infusion diminished after the vagotomy procedure (Figure 4H). In addition, consistent results from immunostaining demonstrated a reduction in c‐Fos and 5‐HT co‐localization, indicating decreased activation of 5‐HT^DRN^ neurons following vagotomy (Figure 4I,J). Our data suggests that manipulation of lung microbiota activates lung‐innervating sensory neurons projecting to the NTS, and that unilateral vagotomy abolishes this activation, ultimately leading to decreased c‐Fos expression and reduced co‐localization of c‐Fos and 5‐HT in the DRN. Collectively, these findings indicate that certain lung microbiota can positively activate pulmonary afferent neurons projecting to the CNS, thereby facilitating their interaction with the CNS.

### 2.4 Activation of 5‐HT DRN Neurons Via the Pulmonary BDNF‐TrkB Pathway

**Figure 5:**
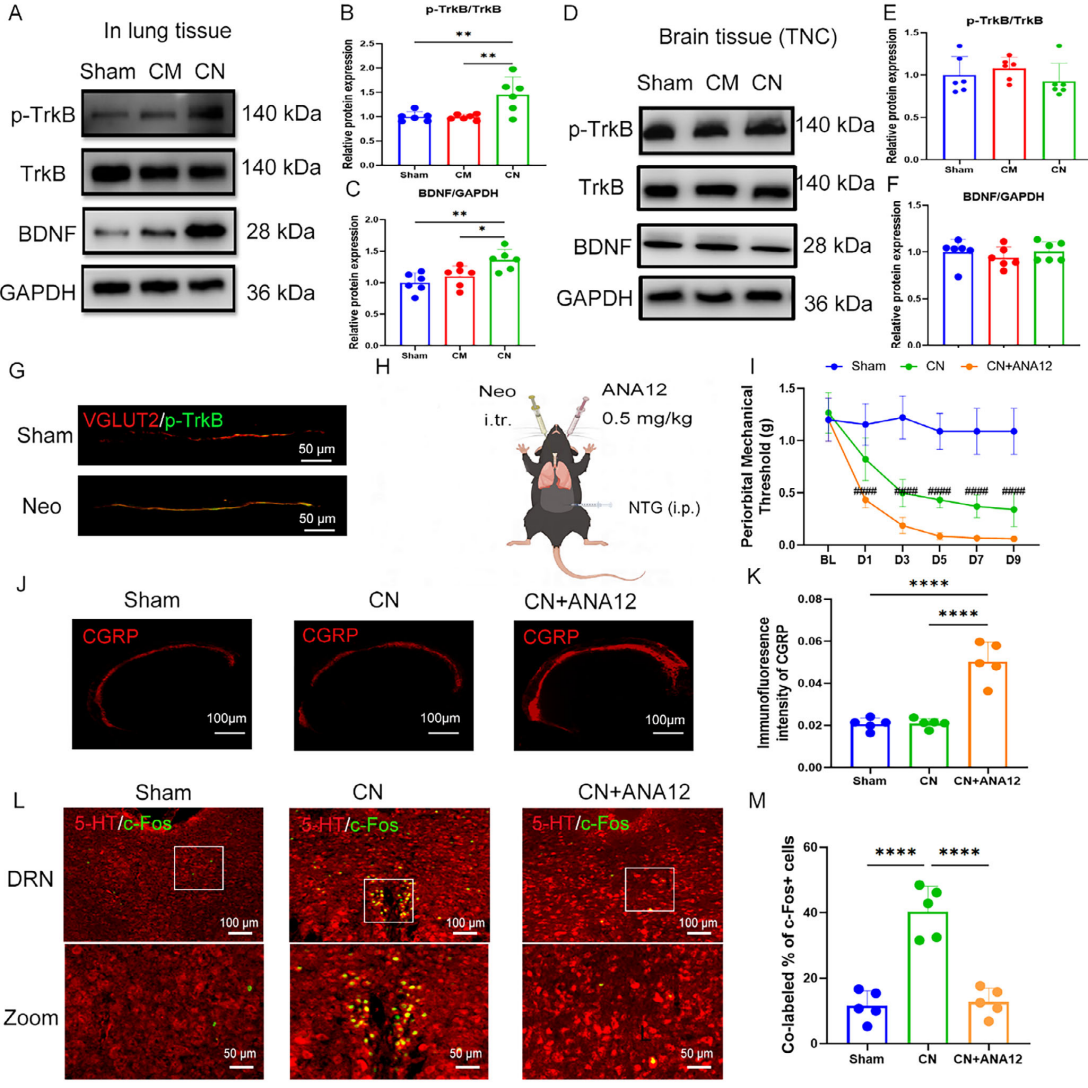
The pulmonary microbiota activates the vagus nerve by activating the BDNF‐TrkB axis in the lungs. A–C) Representative image (A) and quantification (B,C) of western blot showing that neomycin injection increased the level of BDNF and p‐TrkB in the lungs. *n* = 6. D–F) Representative image (D) and quantification (E,F) of western blot showing that neomycin injection did not influence the level of BDNF and p‐TrkB in the TNC tissue (brain). *n* = 6. G) Representative immunofluorescence images showed that neomycin injection in the lungs activated p‐TrkB in VGLUT2‐expressing neurons. Scale bar, 50 µm. *n* = 3. H) Schematic diagram showing the experimental procedures related to ANA12 intratracheal injection of neomycin‐treated chronic migraine (CN) group mice. I) The periorbital mechanical threshold of sham, neomycin‐treated chronic migraine (CN), and CN+ANA12 group. *n* = 5. J,K) Representative images (J) and quantification (K) of CGRP expression in the TNC tissue, Scale bar: 100 µm. *n* = 5. L,M) Representative images (L) and quantification (M) of 5‐HT and c‐Fos colocalization in the DRN. Scale bar: 100 µm. *n* = 5. Values are presented as mean ± SEM. *p < 0.05, **p < 0.01, ***p < 0.001, and ****p < 0.0001. One‐way ANOVA test or two‐way ANOVA test was used for multivariate analysis.

Brain‐derived neurotrophic factor (BDNF) is the only known neurotrophic factor influenced by microbiota,^[^ ^41^ ^]^ prompting us to investigate its role in the regulation of the pulmonary microbiome. We observed an increased expression of BDNF protein in the lungs of the neomycin‐treated chronic migraine group, along with elevated phosphorylation levels of its receptor, tropomyosin receptor kinase B (TrkB) (**Figure** **5**A–C). In contrast, there was no significant change in BDNF or TrkB phosphorylation levels in the brain (Figure 5D–F). In addition, the phosphorylation of TrkB was enhanced in Vesicular Glutamate Transporter 2 (VGLUT2)‐expressing neurons within the lungs (Figure 5G), supporting our hypothesis that alterations in pulmonary microbiota activate the pulmonary BDNF‐TrkB axis, thereby stimulating sensory neurons of the vagus nerve without affecting BDNF‐TrkB signaling in the brain.

To investigate the role of pulmonary BDNF‐TrkB in this process, we administered ANA12, a small molecule inhibitor of TrkB, intratracheally prior to the administration of neomycin (Figure 5H). Our findings revealed that following ANA12 treatment, mice in the neomycin group exhibited significant reductions in the periorbital mechanical threshold, indicating increased pain sensitivity (Figure 5I). Further, immunofluorescence assessments revealed increased CGRP expression in the TNC tissue of ANA‐12‐treated mice (Figure 5J,K). Staining of the DRN region revealed a significant reduction in the colocalization of 5‐HT and c‐Fos following ANA‐12 treatment (Figure 5L,M), indicating decreased activation of 5‐HT neurons. These results suggest that the pulmonary BDNF‐TrkB pathway plays a crucial role in the modulation of migraines associated with pulmonary microbiota.

**Figure 6:**
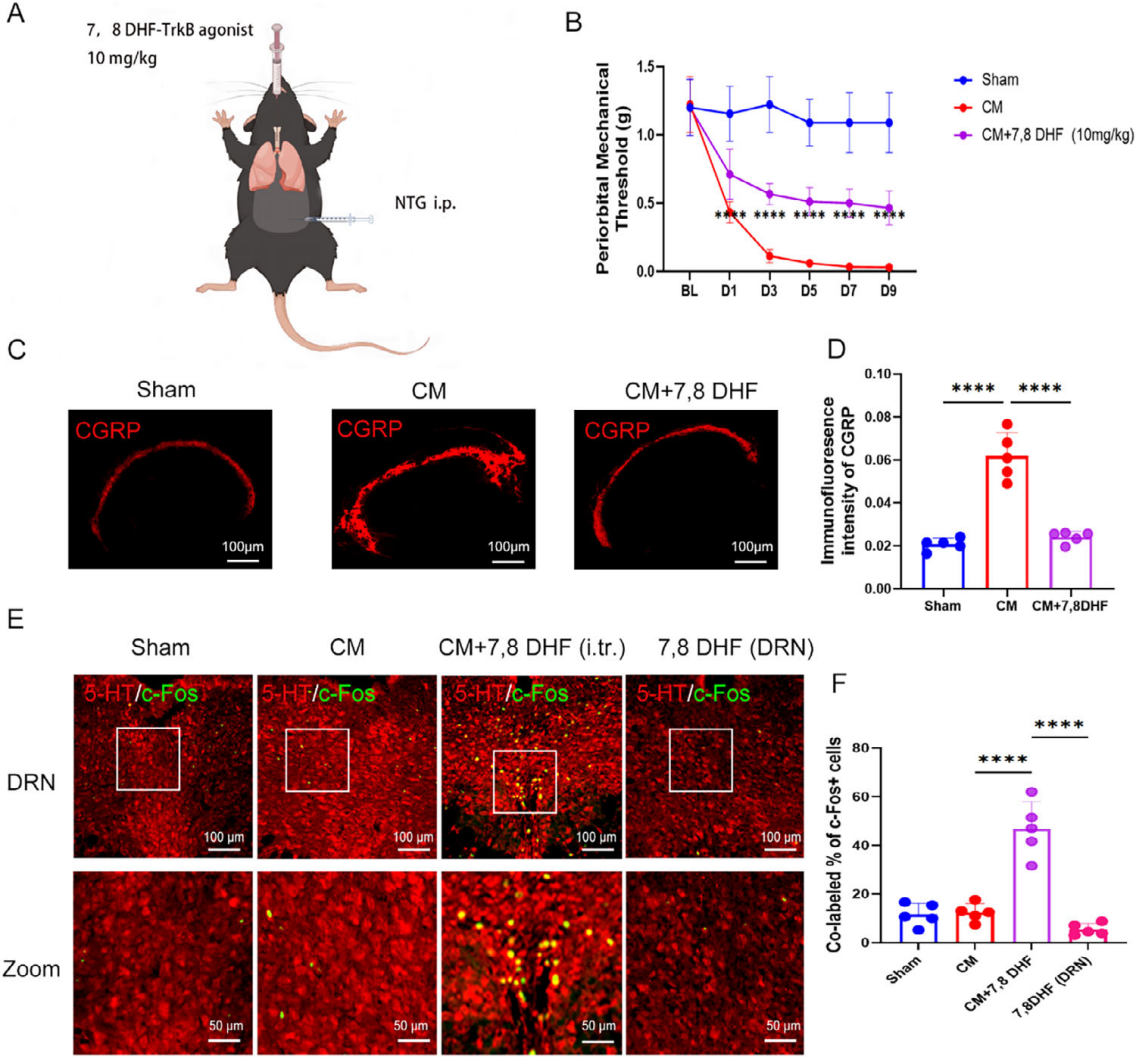
TrkB activation in the lungs via 7,8 DHF alleviates migraine symptoms by activating 5‐HT DRN neurons. A) Schematic of the experimental procedures for 7,8 DHF intratracheal injection in the chronic migraine (CM) group mice. B) The periorbital mechanical threshold of sham, chronic migraine (CM), CM with 7,8 DHF intratracheal injection. *n* = 6 per group. ****p < 0.0001 versus CM. C,D) Representative images (C) and quantification (D) of CGRP expression in the TNC tissue after 7,8 DHF treatment, Scale bar: 100 µm. *n* = 5. E,F) Representative images (E) and quantification (F) of 5‐HT and c‐Fos colocalization after 7,8 DHF intratracheal injection. Scale bar: 100 µm. *n* = 5. Values are presented as mean ± SEM. *p < 0.05, **p < 0.01, ***p < 0.001, and ****p < 0.0001. One‐way ANOVA test or two‐way ANOVA test was used for multivariate analysis.

Based on our findings that the lung microbiome can activate pulmonary vagal afferent nerves through the pulmonary BDNF‐TrkB pathway to regulate migraine, we administered 7,8‐Dihydroxyflavone (7,8 DHF), a TrkB agonist, via intratracheal injection to the migraine group (**Figure** **6**A). Prior to each intraperitoneal injection of NTG for migraine induction, mice received an intratracheal dose of 10 mg kg^−1^ 7,8 DHF or vehicle control. Mice treated with 7,8 DHF exhibited a higher periorbital mechanical threshold (Figure 6B). In addition, there were lower CGRP levels in the TNC (Figure 6C,D) and increased co‐localization of 5‐HT and c‐Fos in the DRN from 7,8 DHF‐treated mice (Figure 6E,F). However, direct injection of 7,8 DHF into the DRN region did not result in similar activation of 5‐HT neurons (Figure 6E,F). These results suggest that intratracheal administration of 7,8 DHF enhances TrkB activation in the lungs, thereby activating 5‐HT neurons in the DRN region to alleviate migraines.

### 2.5 Lung Microbiota Sends Neural Signals Via the Circuit of Pulmonary Sensory Nerves to the DRN

#### 2.5.1 Neural Circuit Tracing Reveals Pulmonary Sensory Pathways to the DRN

**Figure 7:**
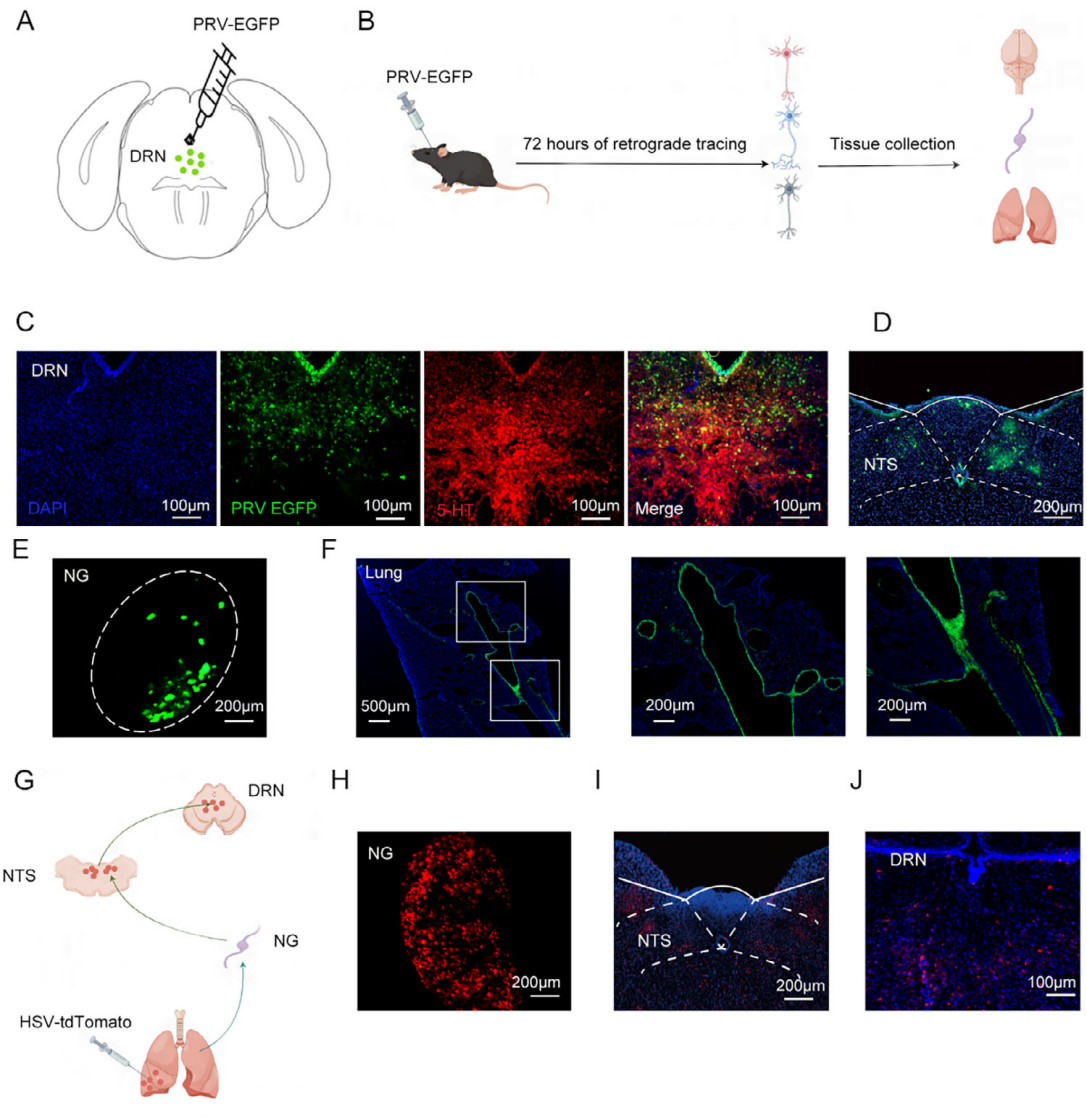
Neural circuit mapping from pulmonary sensory neurons to the DRN. A) Polysynaptic pseudorabies virus (PRV‐GFP) was injected into the DRN (Bregma: – 4.6 mm) for retrograde tracing. B) Schematic diagram showing the experimental procedures. C) Representative images showing PRV‐GFP infected neurons predominantly co‐localizing with 5‐HT neurons in the DRN. Scale bar: 100 µm. D–F) Dense retrograde labeling was observed in the NTS (scale bar: 200 µm) (D), in the nodose ganglion (scale bar: 200 µm) (E), and in the lung (scale bar: 500 µm) (F). G) The polysynaptic herpes simplex virus (HSV‐tdTomato) was injected into the lung. H–J) Representative images showing the HSV‐tdTomato labeling in the nodose ganglion (scale bar: 200 µm) (H), in the NTS (scale bar: 200 µm) (I), and in the DRN (scale bar: 100 µm) (J).

To investigate how pulmonary sensory nerves transmit neural signals to 5‐HT^DRN^ neurons, we employed a retrograde transsynaptic viral tracer by injecting a green fluorescent protein‐expressing pseudorabies virus (PRV) into the DRN region (**Figure** **7**A,B). Following the infection with PRV‐EGFP, bright GFP fluorescence was observed in the neural fibers and neurons of the DRN, predominantly co‐localizing with 5‐HT neurons (Figure 7C). Seventy‐two hours post‐infection, the PRV had retrogradely labeled neurons in the nucleus prepositus, NTS, nodose ganglia, and the lung (Figure 7C–F), mapping the neural circuit from lung afferents to the DRN. To confirm this circuit, we injected anterogradely transported and polysynaptic herpes simplex viruses (HSV‐tdTomato) into the lung (Figure 7G). Seven days post‐injection, HSV‐tdTomato labeling extended to nodose ganglia, NTS, and DRN targets (Figure 7H–J). These results confirmed that neomycin‐induced changes in the lung microbiota send neural signals from peripheral pulmonary sensory neurons to 5‐HT^DRN^ neurons in the brain, establishing a neural circuit that facilitates communication between the lung microbiome and the CNS.

## 3 Discussion

Our research demonstrates that intratracheal injection of neomycin modifies the pulmonary microbiota, which in turn, influences the central nervous system via the pulmonary‐brain axis. This mechanism activates the serotonin (5‐HT) system, resulting in the alleviation of chronic migraine pain. This approach may represent a novel therapeutic strategy for the treatment of chronic migraines in the future.

Regarding migraines, 5‐HT neurons, primarily located in the DRN, project serotonergic fibers to various levels of the trigeminal nociceptive pathway,^[^ ^42^ ^]^ ultimately modulating migraines and mediating bidirectional analgesic effects.^[^ ^43^ ^]^ Agonists of the 5‐HT1B and 1D receptor subtypes, known as triptans, are currently the first‐line treatment options for moderate to severe migraine attacks.^[^ ^44^ ^]^ However, triptans are contraindicated for patients with cardiovascular diseases and uncontrolled hypertension due to their vasoconstrictive effects, which impose certain limitations on their clinical application. Our data demonstrates that in mouse models, interference with the pulmonary microbiota increases serotonin levels in the brain, alleviating migraine pain. Further, both early and mid‐to‐late stages of the mouse migraine model exhibit significant analgesic effects, indicating that the pulmonary microbiota can stably modulate the central nervous system. However, in clinical settings, the use of intratracheal antibiotics may be restricted. Although animal models have shown that low doses of neomycin can induce changes in and regulate the pulmonary microbiota, the implications for clinical patients remain uncertain and warrant further clinical validation.

The DRN region serves as the primary area for 5‐HT neurons and is the main source of serotonergic pathways that are distributed along the trigeminal vascular pain pathway.^[^ ^32^, ^45^ ^]^ Previous studies have demonstrated that direct electrical stimulation of the vagus nerve can activate this area via neural projections, resulting in the activation of 5‐HT neurons.^[^ ^46^ ^]^ These findings underscore the therapeutic potential of selectively activating the DRN region in migraine treatment. This region should be considered in the development of pharmacological interventions for migraines. In summary, our research has identified an ascending pathway that originates in the lungs and ultimately targets the DRN region, offering a novel approach for non‐invasive and straightforward targeted drug treatment for migraines.

We further found that the regulation of the central nervous system by the pulmonary microbiota through the lung–brain axis primarily occurs via the BDNF‐TrkB signaling pathway in the lungs. The BDNF signaling pathway is frequently discussed in studies related to the pathophysiology and mechanisms associated with migraines. For instance, one study reported a significant reduction in BDNF levels among clinical migraine patients.^[^ ^47^ ^]^ Another study indicated that injecting BDNF into the ventrolateral PAG can protect the structure and function of the PAG, providing both anti‐epileptic and analgesic effects.^[^ ^48^ ^]^ Current research identifies BDNF as a target in the pathophysiology and treatment of migraines. In our study, pulmonary BDNF may act as a signal to initiate the activation of vagal sensory nerves, thereby further regulating migraines. In addition, we propose inhalation therapy with TrkB agonists to activate the pulmonary vagus nerve for migraine treatment. Although vagus nerve electrical stimulation has been suggested as a non‐invasive potential treatment for migraines, with some speculative research on its mechanisms, its efficacy remains uncertain and it is less convenient than inhalation drug therapy, which poses fewer risks. Inhalation treatment with TrkB agonists may serve as an alternative to vagus nerve electrical stimulation therapy.

The branches of the vagus nerve in peripheral organs have been shown to regulate the central nervous system in various diseases, including migraines, anxiety, and depression.^[^ ^43^ ^]^ Previous studies indicate that gut microbiota can modulate the vagus nerve present in the intestines, thereby influencing the central nervous system and playing a crucial role in research related to pain and reward pathways.^[^ ^22^, ^49^, ^50^ ^]^ The lungs, possessing the largest surface area in the human body, contain a substantial number of vagus nerve branches. This suggests that the lungs may also modulate the central nervous system in areas such as pain regulation, emotions, and reward mechanisms. However, knowledge about the role of lung microbiota is limited, and it remains unclear whether they influence the central nervous system or the associated regulatory mechanisms. In our study, we found that specific lung microbiota can locally activate the afferent branches of the vagus nerve within the lungs, subsequently stimulating the vagus nerve and modulating the central nervous system through specific neural pathways; thus, alleviating migraines. This discovery could potentially serve as a target for future therapeutic interventions and treatment strategies.

In this article, we discuss the significant role of the central serotonin system, regulated by the pulmonary microbiome, in chronic migraine mice via the vagal lung–brain axis. However, the ascending and descending neural circuits projected by the activated DRN region have not been thoroughly investigated, which will also become the focus of our next research, to deeply explore the neural circuits involved in lung microbiota modulation of migraine through the lung–brain axis in a chronic migraine model. Besides, there may be additional connections between the lungs and the brain. For example, immune cells in the lungs can respond to alterations in the lung microbiome, and microbial metabolites may also exert effects. Considering the potential for multiple mechanisms underlying the lung–brain connections, future research should investigate the involvement of the lung–brain axis in various pathological conditions to identify key signaling molecules or mechanisms.

In conclusion, our research has established a connection between the lung–brain axis and the regulation of the remote central nervous system via the lung microbiota. In addition, we have identified a pathway that alleviates chronic migraines through modulation of the lung–brain axis. We propose an inhalation therapy as a potential treatment for migraines. Future studies should further explore the lung microbiota and its potential role in various pathological conditions.

## 4 Experimental Section

### Animals

All animal studies and experimental procedures were approved by the Animal Care and Use Committee of Shanghai Chest Hospital, Shanghai Jiao Tong University, on February 17, 2023 [permission no. KS(Y)23022].

Male C57BL/6 mice (aged 6–8 weeks) and BALB/c (aged 6 weeks) were purchased from Jiangsu Huachuang Sino Pharma Tech Co., Ltd. TRAP2, Ai9, and Sert‐Cre mice were a gift from Wenjie Zhou's lab of Songjiang Hospital and Songjiang Research Institute, Shanghai Jiao Tong University School of Medicine, Shanghai 201600, China.

All mice were housed 5 per cage under a 12‐h light–dark cycle (light on from 7 a.m. to 7 p.m., humidity between 30% and 70%, temperatures of 20–22 °C), with free access to food and water.

### NTG Administration and Chronic Migraine Model

Nitroglycerin injection was purchased from Beijing Yimin Pharmaceutical Co., Ltd. (Beijing, China). NTG injection was prepared as a stock solution of 5.0 mg mL^−1^. The vehicle control used in these experiments was 0.9% saline. NTG was freshly diluted in 0.9% saline to a series of predefined doses. All injections were administered at a 10 mL kg^−1^ volume. All mice were tested for baseline mechanical threshold responses immediately prior to the intraperitoneal injection of NTG and post‐treatment mechanical threshold 2 h after NTG injection every second day (5 test days total) for 9 days.

### Periorbital Mechanical Threshold Test

The von Frey test was used to examine mechanical sensitivity and the monofilament was applied to the periorbital region caudal to the eyes and near the midline. The investigators were blinded to the experimental groups. Before testing the periorbital sensitivity, the mice were placed into a 9‐cm‐long restraining glass cylinder such that only the head poked out. The restrainer allowed head and forepaw movements but prevented the animals from turning in the cylinder. Then followed the application of a von Frey monofilament to the periorbital area of the face over the rostral portion of the eye. A positive response was defined as the mouse stroking its face with the ipsilateral forepaw, quickly retracting its head from the stimulus, or vocalizing. Each site was tested at least three times with an interval of at least 1 min.

### Antibiotic Treatment

Neomycin (MedChemExpress) was administered intratracheally (daily dose: 0.2 mg; volume: 25 µL). ABX antibiotics treatment contained ampicillin (1mg mL^−1^, Sigma–Aldrich), neomycin (1mg mL^−1^, MedChemExpress), metronidazole (1mg mL^−1^, Sigma–Aldrich), and vancomycin (0.5 mg mL^−1^, Sigma–Aldrich) for 7 days via nebulization (25 mL d^−1^). The antibiotics were freshly prepared before every treatment by dissolving the powder in sterile PBS. An equivalent volume of PBS was used as control. The mice were treated daily for 7 days before starting the experiment. Daily treatment was continued throughout the entire experiment. Of note, the intratracheal antibiotic treatments were well tolerated by the mice with no respiratory distress or diarrhea observed, and the mice steadily increased their body weight comparably to the controls.

### Collection of BALF

BALF was collected under sterile conditions. In brief, the mice were euthanized using an overdose of tribromoethanol (1.25%, Nanjing AIBI Bio‐Technology Co., Ltd). After carefully disinfecting the mouse's fur with 70% ethanol, a tracheotomy was performed and a sterile gavage needle (22G) was inserted into the trachea under a laminar flow hood. Two milliliters of pre‐warmed (37 °C) PBS was slowly instilled into the lung and after 30 s, a volume of 1 mL was retrieved. This step was repeated once, yielding 2 mL of total BALF per mouse. BALF (in 2‐mL aliquots) was immediately snap‐frozen on dry ice and stored at −80 °C until further processing.

### Transfer of BALF‐Derived Microbiota

Mice were intratracheally treated with neomycin. After 7 days, the treatment was stopped and the mice were intratracheally transferred with BALF‐derived microbiota of mice that were intratracheally pre‐treated for 7 days with either PBS or neomycin (donor group). Immediately after the microbiota transfer, CM was induced by intravenous injection of NTG. The treatment with BALF‐derived microbiota was performed daily for the entire disease course.

For the collection of microbiota, sterile BALF (2 mL per mouse) was collected daily from either the PBS‐ or the neomycin‐donor group, as described in “Collection of BALF”. The BALF was centrifuged for 10 min at 13 000 rpm and 4 °C, and the bacterial pellet was resuspended in 75 µL sterile PBS. Each mouse of the recipient group received a daily dose of 25 µL of the corresponding BALF resuspension.

### Stereotaxic Injection

For intracranial injection, mice were anaesthetized with tribromoethanol (1.25%, 0.2 mL 10 g^−1^) and fixed on a stereotaxic device (RWD Life Science) equipped with an electronic heating pad. The scalp was cut and a 10‐mm incision was made posterior to the bregma along the midline. Injections were performed with a Hamilton 10‐µL syringe needle (Hamilton 7000 series) and a micro‐injection pump (MC‐Nano00, Shanghai Meilisai Life Science Co., Ltd.) at a rate of 100 nL min^−1^. After injection, the glass pipette was left at the site for 5 min, and then, slowly withdrawn. The scalp was sutured and the mouse was allowed to fully recover from anesthesia and returned to its home cage. All viral vectors and drugs were subdivided into aliquots and stored at −80 °C until use.

### AAVs for 5‐HT Neuron Elimination

To specifically eliminate the serotonergic neurons in the DRN, a 1:1 mixture of rAAV‐EF1a‐flex‐taCasp3‐TEVp‐WPRE‐hGH pA and rAAV‐TPH2‐CRE‐WPRE‐hGH pA purchased from BrainVTA (Wuhan, China) was injected into the DRN (Anterior‐posterior [AP]: −4.6 mm; Medial‐lateral [ML]: −1.1 mm; Dorsal‐ventral [DV]: −3.2 mm; at a 20° angle lateral to the midline). The injection volume of the AAV mixture per mouse was 300 nL.

### AAVs for hM4Di‐Mediated 5‐HT Neuron Inhibition in DRN Region of Sert‐Cre Mice

To specifically induce the silencing of DRN 5‐HT neurons, rAAV‐EF1a‐DIO‐hM4D(Gi)‐mCherry‐WPRE‐hGH (BrainVTA, Wuhan, China) was injected into the DRN of Sert‐Cre mice. The injection volume of the AAV per mouse was 150 nL. After 2 weeks, the DREADD agonist clozapine N‐oxide (CNO, MedChemExpress) was administrated through the drinking water (25 µg mL^−1^) after chronic migraine model pretreated with neomycin. Mice injected with rAAV‐EF1a‐DIO‐mCherry‐WPRE‐hGH (BrainVTA, Wuhan, China) and CNO were used as the vehicle group.

### PRV‐EGFP for Retrograde Tracing

The mouse was anesthetized by intraperitoneally injecting tribromoethanol (1.25%, 0.2 mL 10 g^−1^) and fixed on a stereotaxic instrument (RWD Life Science). The procedure of stereotaxis injections was described above. PRV‐EGFP purchased from BrainVTA (Wuhan, China) was injected in the DRN (Anterior‐posterior (AP): −4.6 mm; Medial‐lateral (ML): −1.1 mm; Dorsal‐ventral (DV): −3.2 mm; at a 20° angle lateral to the midline) for retrograde projection mapping. The injection volume per mouse was 300 nL.

### HSV‐tdTomato for Anterograde Tracing

The mouse was anesthetized by intraperitoneally injecting tribromoethanol (1.25%, 0.2 mL 10 g^−1^), and a longitudinal midline incision was made in the ventral region of the neck before blunt dissection. A small incision was made on the trachea after full exposure, and HSV‐tdTomato (3 µL) purchased from BrainVTA (Wuhan, China) was slowly injected into the trachea for anterograde projection mapping by a Hamilton 10‐µL syringe needle (Hamilton 7000 series). After virus injection, the microsyringe was pulled out and the incision was closed using a 4‐0 silk suture. 7 days after injection, the brain was collected for fluorescent signal observation.

### Unilateral Vagotomy

A longitudinal midline incision was made in the ventral region of the neck before blunt dissection. The overlying muscles and fascia were separated to reveal the left or right vagal nerves. For the vagotomy group, the vagus was carefully stripped away from the carotid artery and precisely cut. For the sham group, the vagus was kept intact. The wound was closed and sutured.

### Immunofluorescence Staining

Mice were transcardially perfused with PBS followed by 4% paraformaldehyde. Following perfusion, tissues were harvested and kept in 4% paraformaldehyde for 24 h, and then, in 30% sucrose solution for 24–48 h. The tissues were then frozen and cut into 40‐µm sections. All samples were thoroughly rinsed in PBS to remove any residual OCT compound. The tissue sections were incubated with Triton X‐100 (Beyotime, Catalog No. P0097) and blocking buffer (Beyotime, Catalog No. P0260) for 1 h at room temperature, followed by incubation with primary antibodies at 4 °C overnight. The primary antibodies included 1) mouse anti‐c‐Fos IgG, 1:1000, mouse monoclonal (2H2) to c‐Fos, Abcam, Catalog No. ab208942; 2) rabbit anti‐5‐HT, 1:500, Immunostar, Catalog No. 20080; 3) rabbit anti‐CGRP, 1:1000, Cell Signaling Technology, USA, Catalog No. 14959; 4) rabbit anti‐VGLUT2, 1:300, Abcam, Catalog No. ab216463; and 5) rabbit anti‐Phospho‐TrkA+TrkB, 1:250, Bioss, Catalog No. bs‐3457R.

Next, the sections were incubated with secondary antibodies at room temperature for 1 h. Secondary antibodies included 1) goat anti‐mouse IgG Alexa 488, 1:1000, Invitrogen, Catalog No. A‐11001; 2) goat anti‐rabbit IgG Alexa 488, 1:1000, Invitrogen, Catalog No. A‐11008; and 3) goat anti‐rabbit IgG Alexa 594, 1:1000, Invitrogen, Catalog No. A‐11012. In addition, nuclei were counterstained with DAPI (4′, 6‐diamidino‐2‐phenylindole) for 10 min. Images were acquired using a Olympus, VS200 confocal microscope, LEICA DMi8 microscope.

### c‐Fos Measurements

To investigate the effects of intratracheal neomycin injection on neuronal activity in the brain, frozen sections of the brain were obtained, and the expression of c‐Fos was assessed through immunofluorescence to visualize neuronal activation in the DRN and the NTS. To evaluate neuronal activation in the nodose ganglion, mice were sacrificed at the conclusion of the experiment and perfused as previously described. The vagus nerve was carefully separated from the carotid artery until the nodose ganglion was accessible. Neuronal activation was visualized by detecting c‐Fos expression through immunofluorescence in the entire nodose ganglion.

### Enzyme‐Linked Immunosorbent Assay (ELISA)

Mice were anaesthetized with tribromoethanol at the indicated time points. Fresh mouse brains were removed, homogenized in PBS containing protease and phosphatase inhibitors (TargetMol), and centrifuged for 15 mins at 12 000 rpm for supernatant. The supernatant was collected and immediately stored at −80 °C until use. The concentrations of TNF‐α (YEPCOME Biotechnology Co., Ltd, Shanghai, China, BY‐EM220852), IL‐6 (YEPCOME Biotechnology Co., Ltd, Shanghai, China, BY‐EM220188), IL‐1β (YEPCOME Biotechnology Co., Ltd, Shanghai, China, BY‐EM220174), and 5‐HT (YEPCOME Biotechnology Co., Ltd, Shanghai, China, BY‐JZF0046) were then analyzed using the enzyme‐linked immunosorbent assay (ELISA) kits according to the manufacturer's specifications. Finally, the cytokine concentration was measured according to the absorbance at 450 nm by using a Microplate Reader.

### Agonist and Antagonist Intratracheal Treatment

To target the lungs, the TrkB antagonist, ANA12 (0.5 mg kg^−1^), and the TrkB agonist, 7, 8‐DHF (10 mg kg^−1^), were delivered directly into the trachea. 7, 8‐DHF was purchased from Selleck and prepared in a vehicle of 17% dimethylsulfoxide in PBS. ANA‐12 was purchased from Selleck and prepared in a vehicle of 1% dimethylsulfoxide in physiological saline.

### Western Blot Assays

Fresh mouse lungs and brain tissues were collected for western blot analysis. Tissues were homogenized in RIPA lysis buffer (Solarbio) containing phosphatase and protease inhibitor cocktails (TargetMol) and then centrifuged at 12 000 rpm for 20 min at 4 °C. Supernatants were collected and immediately stored at −80 °C until use. Proteins were separated by SDS‐PAGE, transferred to a polyvinylidene fluoride (PVDF) membrane, blocked with 5% non‐fat milk, incubated overnight with the appropriate primary antibody at 4 °C, and then, incubated with secondary antibodies at room temperature. Specifically, the primary antibodies included mouse anti‐BDNF, (1:5000, ABclonal, Catalog No. A18129), rabbit anti‐TrkB (1:10 000, ABclonal, Catalog No. A21227), rabbit anti‐Phospho‐TrkA+TrkB, (1:1000, Bioss, Catalog No. bs‐3457R) and mouse anti‐GAPDH (1:200 000, ABclonal, Catalog No. AC033). Secondary antibodies included anti‐rabbit IgG, HRP‐linked antibody (1:2000, Cell Signaling Technology, Catalog No. 7074 S) and anti‐mouse IgG, HRP‐linked antibody (Cell Signaling Technology, Catalog No. 7076 S). Immunoreactive proteins were visualized using enhanced chemiluminescence (ChemiDoc XRS1, Bio–Rad, Hercules, CA). The intensities of the light‐emitting bands were detected and quantified with Image Lab software (ImageJ, version 2.0.0‐rc‐69/1.52p). The expression levels of BDNF were normalized to the expression level of GAPDH. The levels of p‐TrkB were normalized to the level of the total form of TrkB.

### 16S DNA Extraction and Amplification

Total genomic DNA was extracted using MagPure Soil DNA LQ Kit (Magan) following the manufacturer's instructions. DNA concentration and integrity were measured with NanoDrop 2000 (Thermo Fisher Scientific, USA) and agarose gel electrophoresis. Extracted DNA was stored at –20 °C until further processing. The extracted DNA was used as a template for PCR amplification of bacterial 16S rRNA genes with the barcoded primers and Takara Ex Taq (Takara). For bacterial diversity analysis, V3–V4 variable regions of 16S rRNA genes were amplified with universal primers 343F (5′‐TACGGRAGGCAGCAG‐3′) and 798R (5′‐AGGGTATCTAATCCT‐3′) for V3–V4 regions.

### 16S Library Construction and Sequencing

The Amplicon quality was visualized using agarose gel electrophoresis. The PCR products were purified with AMPure XP beads (Agencourt) and amplified for another round of PCR. After purification with the AMPure XP beads again, the final amplicon was quantified using Qubit dsDNA Assay Kit (Thermo Fisher Scientific, USA). The concentrations were then adjusted for sequencing. Sequencing was performed on an Illumina NovaSeq 6000 with 250 bp paired‐end reads (Illumina Inc., San Diego, CA; OE Biotech Company; Shanghai, China).

### Statistics and Reproducibility

The data are expressed as the mean ± SEM. The ImageJ and GraphPad 9.0 software programs were used to organize the data, generate the figures, and perform the statistical tests. All behavioral tests and other experiments were performed in a blind manner. Two‐way repeated‐measures ANOVA followed by Tukey's post hoc test was used to analyze behavioral data. For the other data, comparisons between two groups were made using two‐tailed unpaired Student's *t‐*tests, and comparisons among more than three groups were made using one‐way ANOVA followed by Tukey's post hoc test. Detailed information on statistical tests and sample sizes is indicated in the Figure legends, *n* indicates mouse number. All representative micrographs are from three independent experiments. *P < 0.05, **P < 0.01, ***P < 0.001, ****P < 0.0001, and ns – no significant difference.

### Ethics Approval and Consent to Participate

The study protocol was approved by the Animal Care and Use Committee of Shanghai Chest Hospital, Shanghai Jiao Tong University, on February 17, 2023 [permission no. KS(Y)23022]_._

## Conflict of Interest

The authors declare no conflict of interest.

## Author Contributions

B.L. pioneered and designed the study, carried out the experiments, and analyzed the data. C.H., X.L., and H.Y. carried out histological and immunological experiments. Y.X. and K.L. helped with data analysis. J.W. designed the study and analyzed the data. B.L., X.Y., and J.W. wrote the paper.

## Supporting information



Supporting Information

## Data Availability

The data that support the findings of this study are available in the supplementary material of this article.
