# Supplementary material for: The Lung Microbiome Modulates Pain‐Like Behavior Via the Lung–Brain Axis in a Nitroglycerin‐Induced Chronic Migraine Mouse Model
Source: Adv Sci (Weinh). 2025 Mar 31;12(23):2416348. doi: 10.1002/advs.202416348 (PMC12199378; doi:10.1002/advs.202416348)
Supplement: Supplementary file 1 — Supporting Information [file ADVS-12-2416348-s001.docx]

**Supplementary Figure 1.**


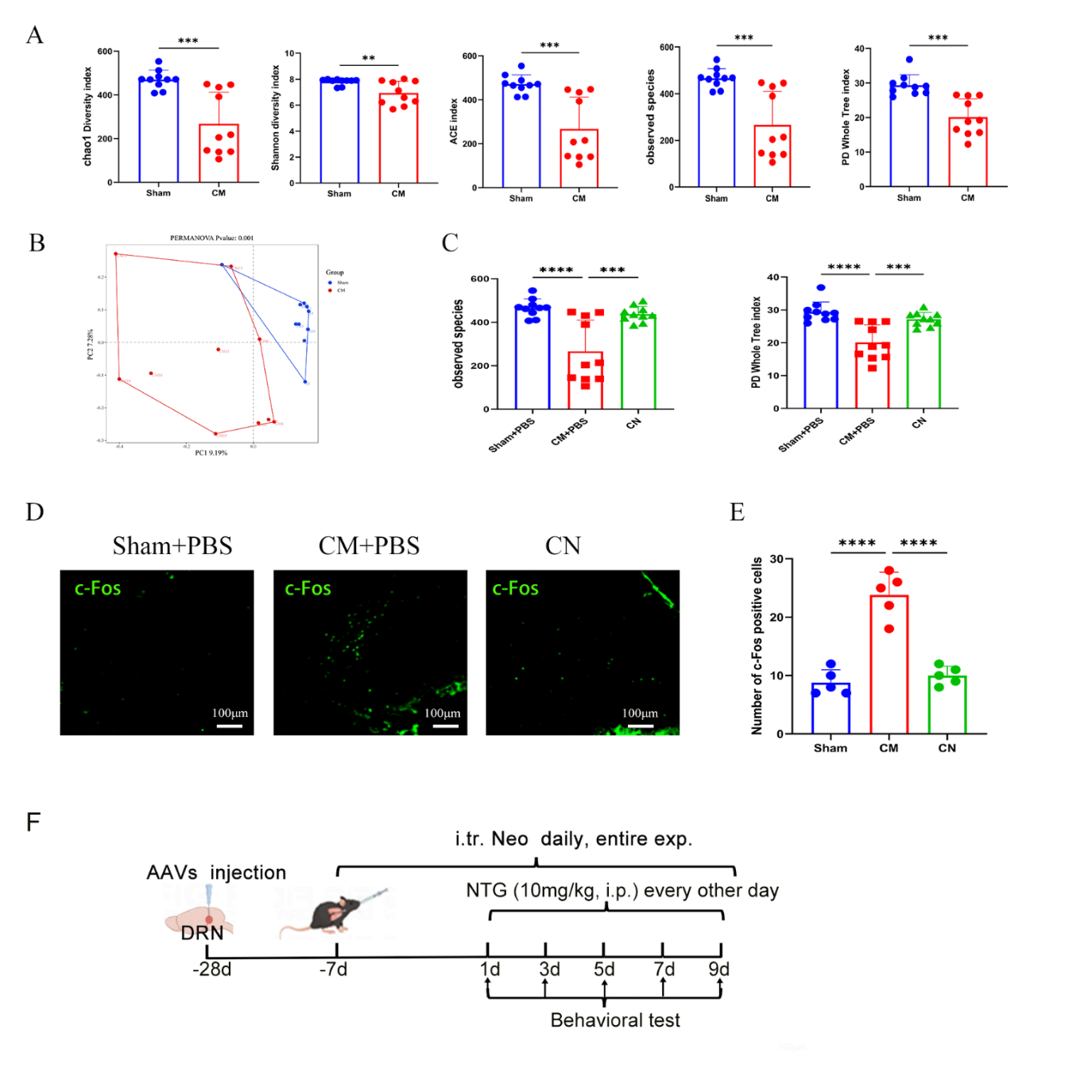


**Supplementary Figure 1.** Supplementary information of microbial diversity and immunostaining. **A** chao1, Shannon, ACE, observed species and PD whole tree index of Sham and CM groups (*n* = 10). **B** Principal co-ordinate analysis of Bray distance (Anosim, *p*=0.001). **C** observed species and PD whole tree index in Sham, CM and neomycin-treated migraine group (CN) groups (*n* = 10). **D-E** Representative images and quantification of c-Fos expression in the TNC tissue after neomycin treatment. Scale bar, 100 µm. n=5. **F** Schematic of the experimental timeline. Values are presented as mean ± SEM. **p* < 0.05, ***p* < 0.01, ****p* < 0.001, *****p* < 0.0001. One-way ANOVA test was used for multivariate analysis. Unpaired t-tests were used for the comparison of two groups.

**Supplementary Figure 2.**


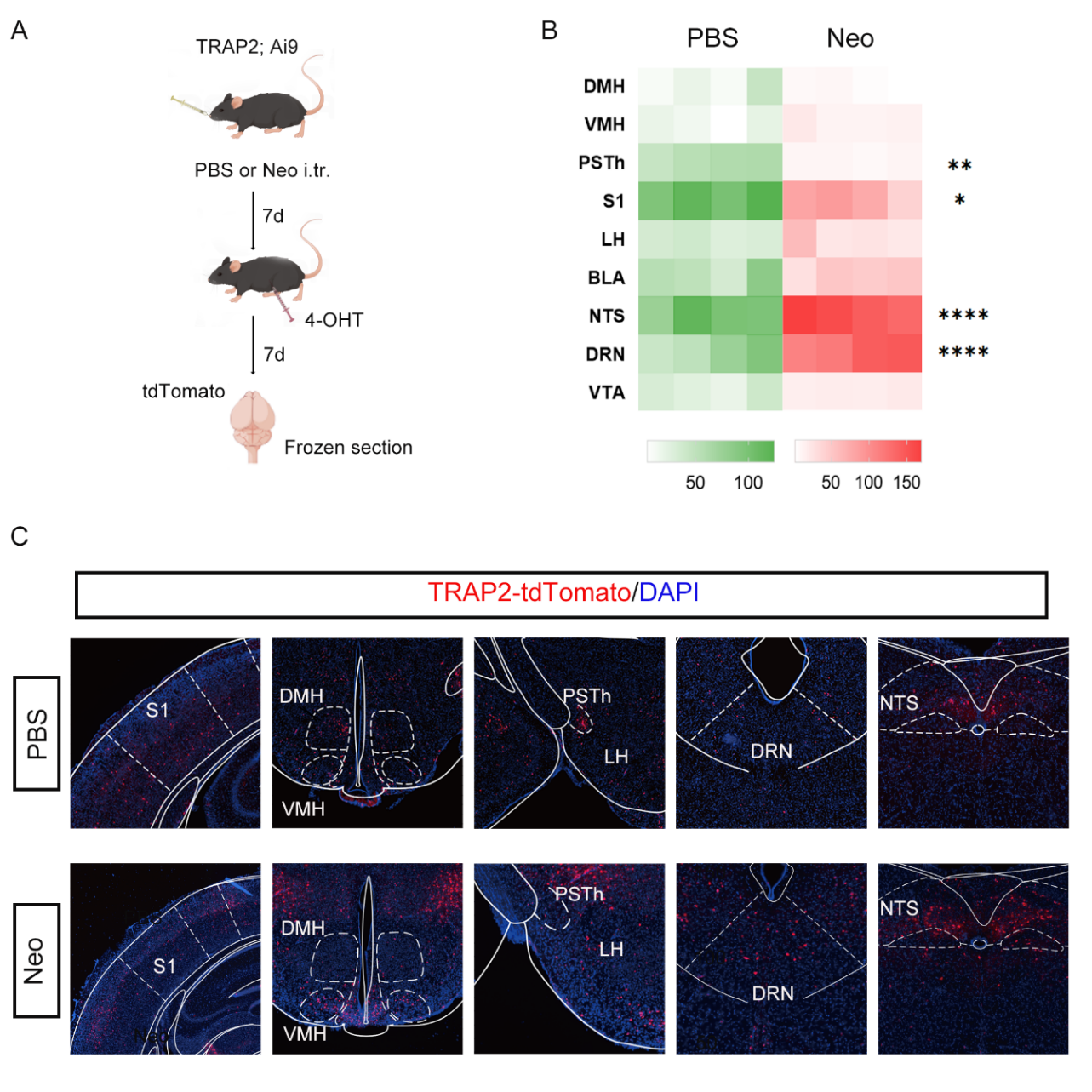


**Supplementary Figure 2.** The activation of different brain regions in PBS or neomycin injection group of TRAP2; Ai9 mice **A** Schematic of the experimental timeline. 4-OHT, 4-Hydroxytamoxifen. **B** Heatmap illustrating the changes of the TRAPed- tdTomato neurons labeling in the S1, DMH, VMH, PSTh, LH, DRN and NTS. **C** Representative images of the TRAPed- tdTomato neurons labeling in the S1, DMH, VMH, PSTh, LH, DRN and NTS. Unpaired t-tests were used for the comparison of two groups.

**Supplementary Figure 3**


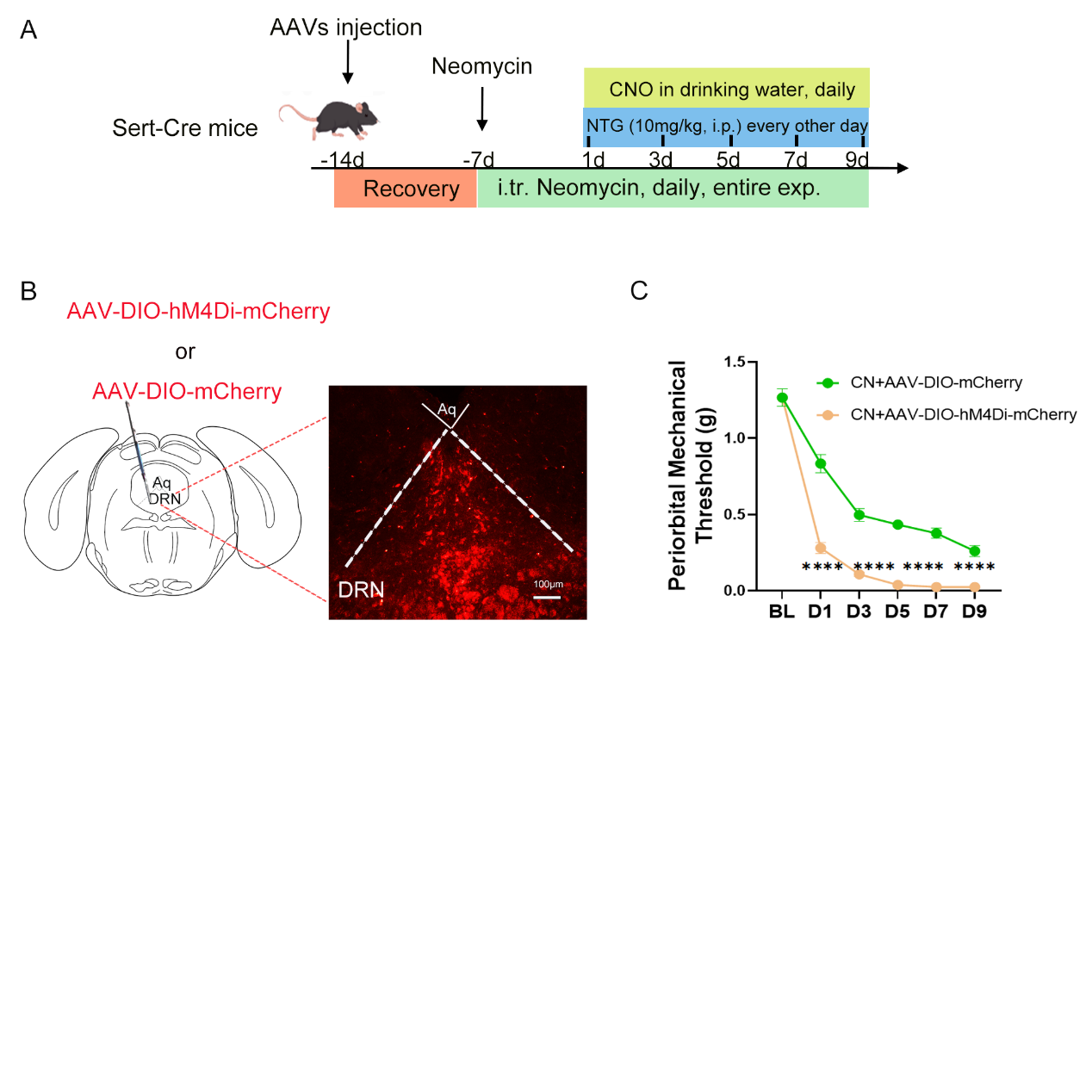


**Supplementary Figure 3: AAV-hM4Di-mediated 5-HT+ neuron silencing inhibited analgesic effects of neomycin treatment.**

**A** Schematic of the experimental timeline. **B** Immunofluorescence image of the injection site in DRN with AAV-DIO-hM4Di-mCherry or AAV-DIO-mCherry. **C** The periorbital mechanical threshold of neomycin-treated migraine mice with hM4Di-mediated 5-HT+ neuron silencing compared to AAV-mCherry. Scale bar, 100 µm (n = 4). Values are presented as mean±SEM. **p* < 0.05, ***p* < 0.01, ****p* < 0.001, *****p* < 0.0001. Unpaired t-tests were used for the comparison of two groups.
